# Evaluation of the response to treatment and clinical evolution in patients with burning mouth syndrome

**DOI:** 10.4317/medoral.18142

**Published:** 2012-12-10

**Authors:** Eugenia Rodríguez-de Rivera-Campillo, José López-López

**Affiliations:** 1 DDS, PhD, Medical Doctor, Doctor Specialist in in Dermatology, Dentist. Adjunct Doctor of Dermaotologyst Sagrat Cor Hospital, Barcelona, Spain. Professor of Oral Medicine at the School of Dentistry, University of Barcelona. Spain; 2 DDS, PhD, Medical and Surgery Doctor. Doctor Specialist in Stomatology. Professor of Oral Medicine at the School of Dentistry, University of Barcelona. Spain; 3 ….

## Abstract

Objective: the aim of this study is to investigate the clinical evolution, the spontaneous remission of the symptomatology and the response to different treatments in a group of burning mouth syndrome patients. Study Design: the sample was formed by a group of patients that were visited in the Unit of Oral Medicine of the Dentistry Clinic of the University of Barcelona, from the year 2000 to 2011. After revising the clinical records of all the patients that had been under control for a period of time of 18 months or longer, they were contacted by telephone. In the telephone interview, they were questioned about the symptomatology evolution and the response to the treatments received, noting down the data in a questionnaire previously performed. Results: the average duration of the symptoms was 6.5 years (+/-2.5 years). The most frequent treatments were: chlorhexidine mouthrinses, oral benzodiazepines, topical clonazepam, antiinflamatory drugs, antidepressants, antifungicals, vitamins, psycotherapy, salivary substitutes and topical corticoids. The specialists that were consulted with a higher frequency were: dermatologists (30%), othorrynolaringologists (10%) and psychiatrists (3%). In 41 patients the oral symptoms did not improve, 35 reported partial improvements, 12 patients worsened, and only in 3 patients the symptoms remitted. Conclusions: In three of the 91 patients studied the symptoms remitted spontaneously within the five years of treatment. Only 42% of the study population had improved the symptomatology significantly, and this improvement would reach 60% if clonazepam were associated to psychotherapy.

** Key words:**Burning mouth syndrome, stomatodynia, oral pain, clonazepam.

## Introduction

Burning mouth syndrome (BMS), also named glossodynia, stomatodynia or stomatopyrosis, is a disease that, even though it has been described for many years, still entails problems when referring to diagnosis and treatment (1-4). It is characterized by the presence of burning or stinging in any area of the oral mucosa, especially on the tongue and lips, with no type of lesion that could justify such symptoms. This disease alters significantly the quality of life of the patients suffering from it (5). The profile of these patients is that of a postmenopausal woman, who would frequently suffer from anxiety and/or depression. Nowadays, there is the debate whether the psychological alterations that they undergo are the cause or the consequence of this chronic pain. The profile of these patients is particular: aged between 50 and 60, they have been suffering aches for a long time, and have been visited and treated by different specialists, without obtaining any solution to their problem. It is usually accompanied by an important emotional profile and is often related to cancerofobia (6). Apart from the oral stinging, other sensitive alterations can take place: a feeling of dryness or gustative alterations – perception of bitter or metallic taste- (7). In some cases they also present other disestesias in the mouth, such as the feeling of sand or swelling (7,8).

This disease has a high prevalence, which varies depending on the studies from 0.7% to 15% (8). Nevertheless, on many occa-sions, these patients go from one specialist to another, with no answer to their problem. This unleashes a bigger anxiety, since they feel misunderstood. As a matter of fact, there are many health practitioners, both in hospitals and private practices that do not provide a successful treatment for these patients, considering them as a burden, and being thankful every time they find another specialist interested in their management. The use of antidepressants, anxiolytics, hypnotics and other xerostomizing –such as antihypertensive drugs and diuretics- may aggravate the feeling of dryness (9-14). People that suffer from this disease usually complain about the dry mouth. However, most of the studies that measure the saliva have not been able to demonstrate or prove quantitative alterations, although in some cases succeeded in proving qualitative alterations (9,14,15).

Although there are many studies published in the literature, the true aetiopathogeny of this disease still remains unknown, which hinders the advancement of the investigation of a treatment that totally effective (10,11,13,16). Some authors treat these symp-toms with capsaicin (17), some others with acid alpha-lipoic (alone or associated to gabapentin) (18,19), with antidepressants or benzodiacepines, such as clonazepam (7,20-22). The clinical evolution of this disease is usually chronic, alternating periods of exacerbation of the symptomatology and periods of improvement. In some cases, spontaneous remissions have been described (23).

Based on the hypothesis that in a small percentage of patients the symptoms return spontaneously 5 years from the beginning, we consider performing a study with the objective of investigating the clinical evolution, the spontaneous remission of the symptoms and the response to different treatments, in a group of patients with BMS.

## Material and Methods

Sample: All the patients visiting the Oral Health Unit of the Odontology faculty of the University of Barcelona from January 2000 to May 2011 were examined meticulously with the objective of ruling out other underlying diseases. It was measured the saliva flow and performed a blood analysis in order to evaluate complete blood cell counts, blood glucose levels, serum iron and transferrin levels, serum vitamin B 12 and folate levels. It was also performed a mycological culturing with the aim of ruling out oral candidiasis. In the cases where it was suspected an allergy related to the contact with any material of the dental prosthesis, “pach tests” were requested. The clinical history of all patients was also reviewed. A total of 184 medical records were revised, and only the records of the patients with BMS of over 18 months of evolution were selected to be part of the study. In the medical record of the patients, all the data obtained from the oral exploration and the blood test was registered. It was also registered the pathologic background of the patient, drugs taken, the clinical characteristics of the stinging, the time of evolution, the different treatments received, and the clinical response.

Out of the 184 revised medical records, only 91 patients were considered for the study –with a time evolution of over 18 months-. Were excluded from the study 65 patients with less than 18 months evolution, 13 patients which did not attend to the controls and 5 patients that were not located. These 91 patients were interviewed by telephone in order to obtain information in relation to their illness and the treatments received.

Methods: in order to register the answers from the tele-phone interviews, a questionnaire was designed ( Table 1). In this docu-ment, all the information referring to the prescribed treatments (either by our group, by any other professionals, or by self-prescriptions) was registered, as well as the evolution of the symptoms, especially the stinging, as a result of the different treatments.

**Table 1:**
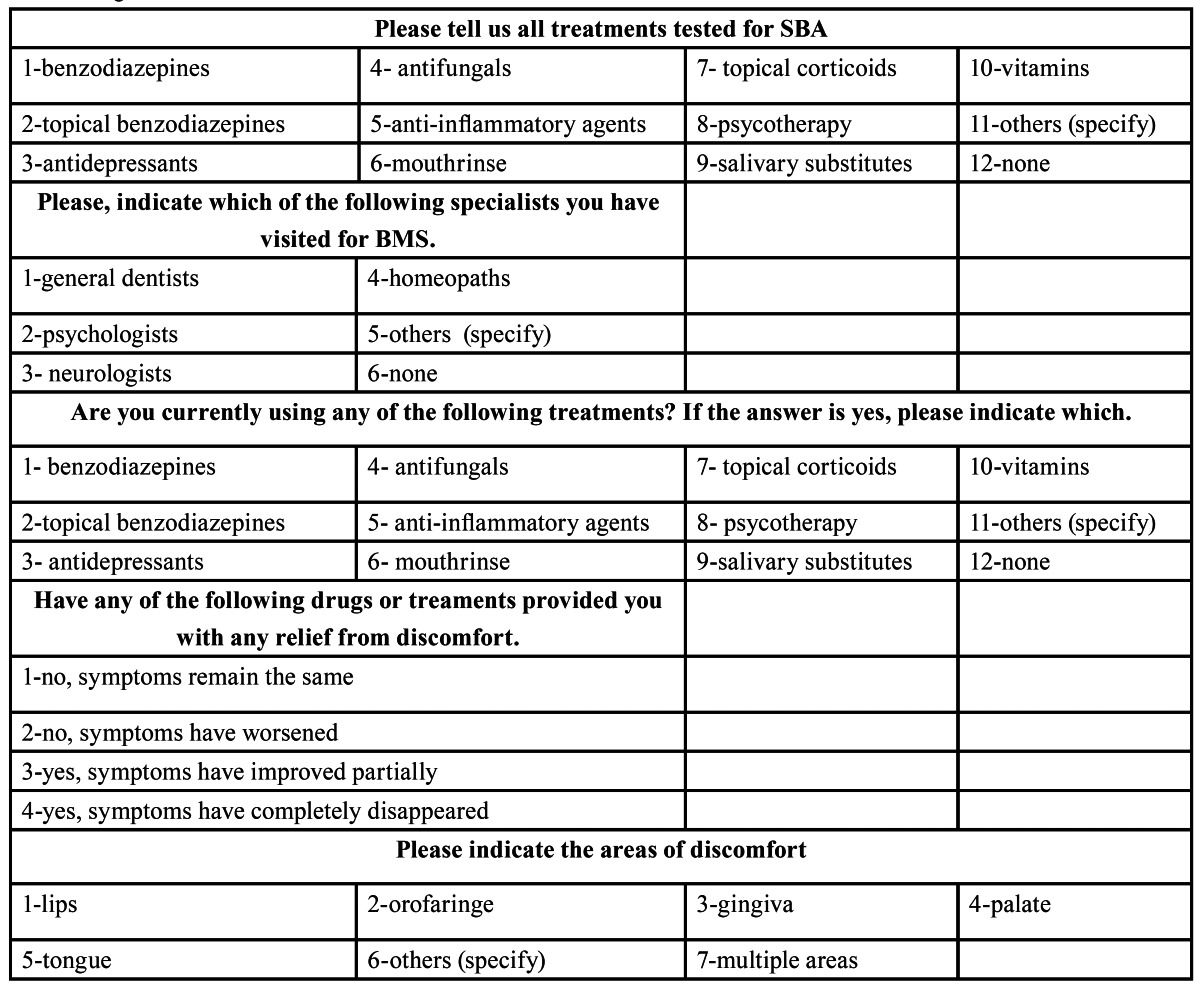
Registration form.

## Results

**Figure 1:**
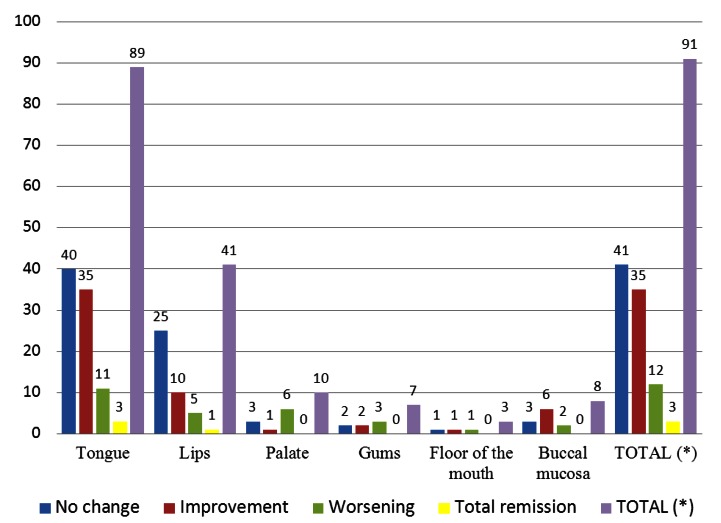
Data referring to the clínical situation of the patients. (*) It refers to the total for each of the clinical behaviours. Bear in mind that every patient is usually under more than one treatment at any one time.

Out of the 91 patients suffering from BMS of >18 months of evolution, 85 were women and 6 were men. The average age was 69.9 years (from 40 to 85 years). The average duration of the symptoms was 6.5 years (+/-2.5 years), and the time during which the patients were managed was 18 to 94 months (7.83 years), with the average at 54 months. The clinical characteristics are summarized in figure 1. Furthermore, the outstanding statistics show that 87 (97.8%) of the patients experienced stinging on the tongue and 41 (45%) on the lip, with a marked preponderance on the lower lip (39 of the 41).

-Therapies

**Figure 2:**
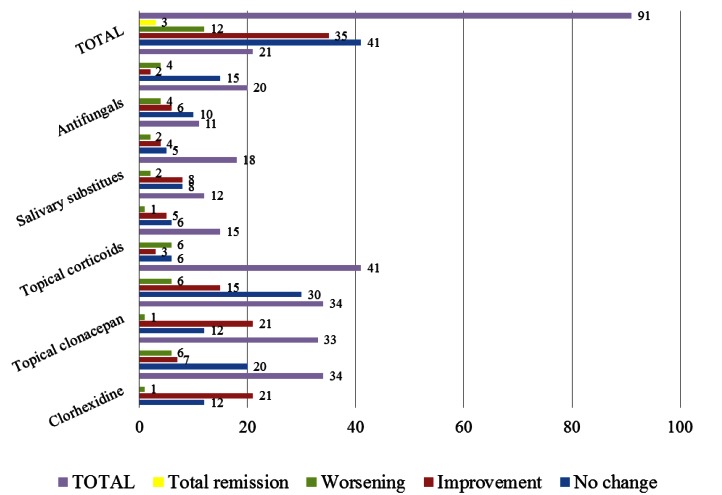
Correlation between symptomatology and the medication used. (*) It refers to the total for each of the clinical behaviours. Bear in mind that every patient is usually under more than one treatment at any one time.

Common treatments used by patients with oral burning symptoms include mouth wash of chlorhexidine, oral benzodiazepines, topical clonazepam, mouth wash, topical corticoids, psychotherapy, salivary substitutes, vitamins, anti-fungicals, and other treatments such as anti-inflammatory drugs or anti-depressants. 32 patients (35% of the sample) had already started some sort of treatment, but they quit after a few days without improvements. The summary of treatments considered in our analysis is listed in figure 2. As shown, the most common treatments were: oral benzodiazepines (36.3%), topical clonazepam (37.4%), antifungicals (21.9%), mouth wash (56.1%) and others such as anti-inflammatory and anti-depressants (23.08%). The summary of the evolution of patients using these five treatments either on its own or in conjunction with other treatments is listed in  Table 2. In addition, pair-wise correlations between types of evolution for each of these five treatments are listed in  Table 3. Moreover, the summary of the evolution of patients using different combinations of these common treatments is listed in  Table 4.

**Table 2:**
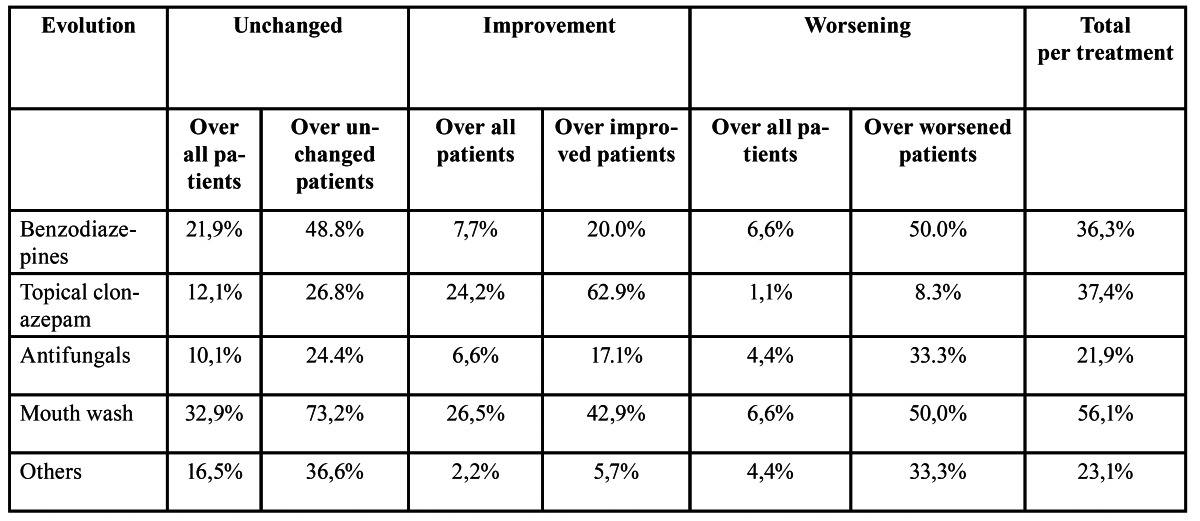
Degree of occurrence for the most common treatments. Statistics include the percentage over the dataset (i.e., ‘unchanged over all patients’ is the percentage of unchanged patients over the total number of patients) and also over the patients with a specific evolution (i.e., ‘unchanged over unchanged’ refers to the percentage of unchanged patients using an specific treatment over the total number of unchanged patients). Last column refers to the percentage of patients using the specific treatment.

**Table 3:**
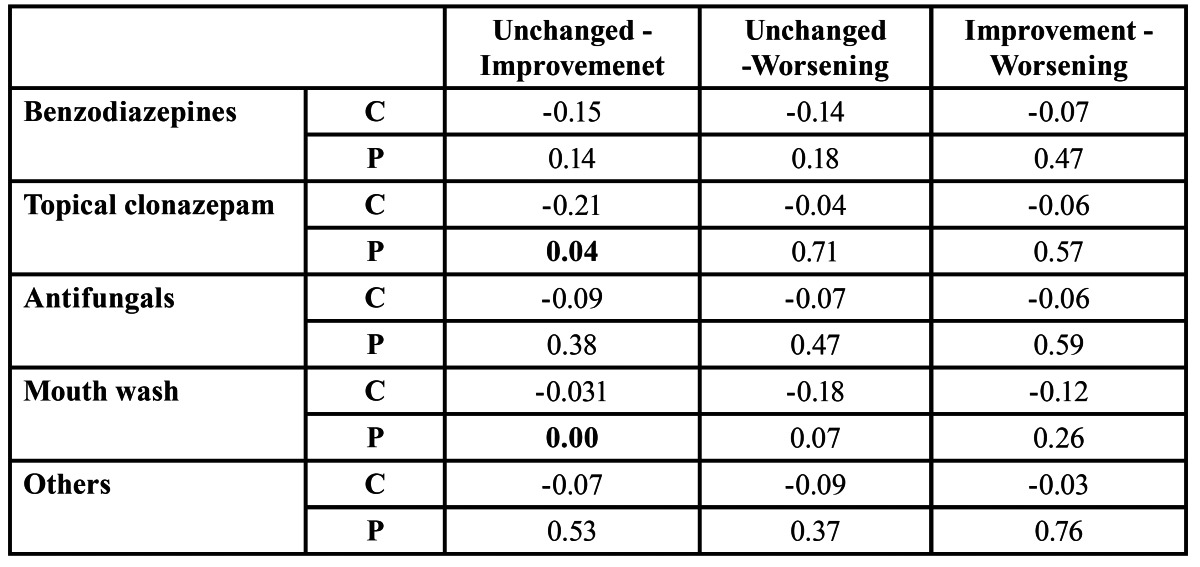
Degree of correlation between the most common treatments independent of the combinations. C stands for pairwise correlation coefficient and P stands for Pearson coefficient. There is a significant correlation between two populations if P<0.5.

**Table 4:**
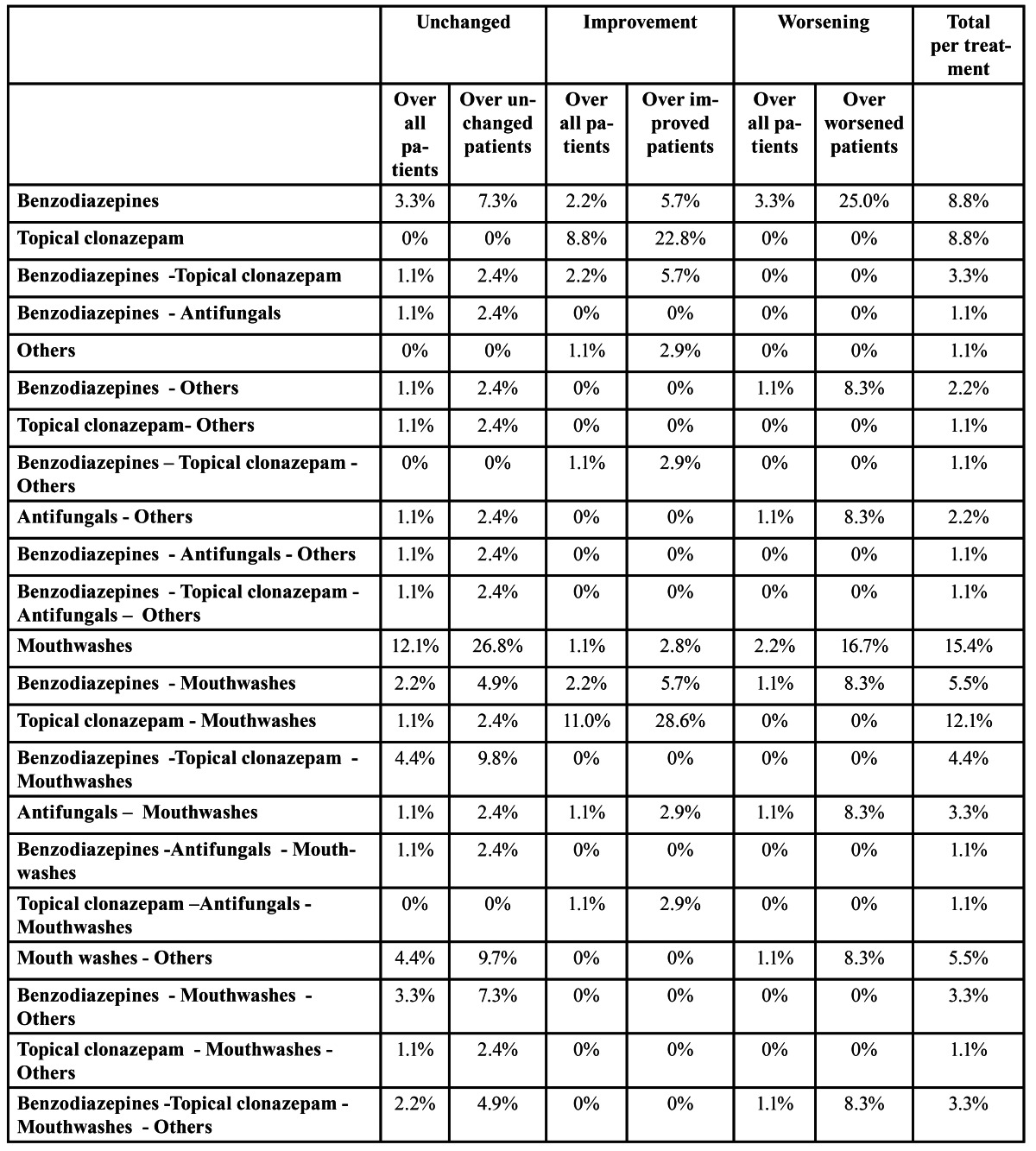
Degree of occurrence for different treatment combinations of the most common treatments. See table 2 for details.

As shown, 37% (34 out of 91) of the patients used topical clonazepam and 24% of these patients felt an improvement in their condition. This represents the 62% of patients improving their condition. That is, more than a half of patients improving their condition used topical clonazepam in their treatment. Main improvements are in patients using topical clonazepam on its own (22,8%) and topical clonazepam combined with mouthwashes (28.6%) as listed in  Table 4. As listed, treatments considering only mouthwash led to no improvement in patient’s condition. However, the combination of mouthwashes with topical clonazepam resulted in an improvement on more than 26 patients. Furthermore, only 12.1% and 1.1% of the patients felt no improvement (unchanged) or deterioration (worsening) respectively when topical clonazepam was included in their treatment. Moreover, there is not significant correlation between pairwise comparisons of patient’s evolution using treatments including topical clonazepam ( Table 3). Hence, from these results, we can conclude that including topical clonazepam improves the condition of a patient presenting oral burning symptoms.

Similarly, 36.3% (33 out of 91) of the patients used oral benzodiazepines in their treatments ( Table 2). However, only 7.7% of them felt an improvement in their condition. In this case, most of the patients felt no changes (21.9%) or worsening (6.6%). This represents the 48.8% of patients feeling no changes and 50.0% of patients feeling worse, respectively. In addition, treatments considering only oral benzodiazepines resulted in worse symptoms in 25% of the patients ( Table 3). Furthermore, there is not significant correlation between comparisons of patient’s evolutions when including oral benzodiazepines. Hence, from these results, we can conclude that the use of oral benzodiazepines does not improve the condition of patients presenting oral burning symptoms.

-Other specialities

74 of the patients visited different specialists (i.e., dermatologists (30%), othorhinolaryngologist (10%), psychiatrists (3%), psychologists (2%), neurologists (2%), homeopaths (1%) and rheumatologists (0.5%)) for alternative oral burning treatments. In particular, 45 patients (49.45%) visited more than one specialist and 13 patients (14.25%) visited up to four specialists. Finally, 16 of these patients visited other dentists.

-Evaluation of the stinging

As shown in figure 2, in 41 patients (48%) the oral symptoms did not improve, 35 (38%) referred partial improvements, 12 (13%) worsened and in only 3 patients (3.2%) the symptoms remitted spontaneously. For the most part, these patients had been under different therapies which had been withdrawn due to the little success they experienced. These 3 patients were women. In one of them, the symptoms disappeared after an 18-month evolution, for unknown reasons. In the other two, symptoms disappeared when the associated anxiety unleashed by family issues was resolved, one of them after 20 months of evolution and the other after 22 months. The 41 patients that had not improved had consulted with an average of 4 professionals, compared to the average of 2 professionals that the 91 patients visited. Out of the 41 patients that did not display any improvement, 12 (13%) had used topical clonazepam with or without other treatments, while 21 of the 35 patients that did show improvements had used it, resulting in a statistical significance (p<0,00). Of the 12 patients that worsened, one of them had used, among other treatments, topical clonazepam.

## Discussion

Although BMS aetipathology mechanisms still remain unknown, it seems pretty obvious that BMS disease is closely related to psychological alterations. Nevertheless, it has not yet been scientifically proven. Recent investigations have related this disease with neurological alterations in the somatosensory branch of the Trigeminal Nerve (24). In that work, the authors suggest that the alterations in the peripheral nervous system, especially in the sensitive branch of the Trigeminal, having found out these alterations in the pupil reflex (25). Other recent studies report improvements in BMS symptoms using acid alpha-lipoic acting as a neuroprotector (18,19), or using topical clonazepam, a drug of neurotropic action (20). Or even clonazepam binding topic in systemic (26). These results reinforce the promising hypothesis that the aetiopathogeny of the BMS intercedes in a peripheral neurological alteration. There are several studies analyzing the effects of different BMS treatments (10,11,13,17). However, currently, there is no completely effective treatment for BMS.

Gruskha et al. (27) in a review presented in 1991 revised the clinical course of 43 patients affected by BMS during a period of time of approximately 6 years. After completing a telephone interview, 23 reported they had not felt any changes. 13 reported complete healing and 7 reported a partial improvement in all the symptoms. Out of the 13 patients that presented a total remission in the symptoms, 9 were spontaneous, and did not require any kind of treatment, corresponding to 20% of the patients. In our population group, 48% of the patients did not experience any improvement, this number is similar to the data presented by Grushka et al (27). In their study, 54% of the patients reported not feeling any changes in the clinical evolution. In our group, we found a higher number of patients with a partial improvement and a lower number with total remission. Only 3 patients experienced complete remission of the symptomatology.

In another study regarding the clinical characteristics of the BMS, Danhauer et al. (28) recounted the spontaneous remission of the symptoms in 1 of the 26 patients (3.8%). The study population comprises of 26 patients with the average age being 59.08 years, +/-12.4 years, and the average duration of the symptoms 2.27 years +/- 3.81 years. We have found spontaneous remission in 3 of 91 patients (3.2%).

More recently, in 2006, Sardella et al. (23), published a study with 53 BMS patients (48 women and 5 men) with an age range from 33 to 82 years (average age of 67.7 years). The average duration of the symptoms was 5.5 years+/- 1.9 years and the average follow-up period was 56 months. As a direct consequence of different fulfilled treatments, 26 patients (49%) did not notice any change in oral symptoms. 15 patients (28.3%) related some sort of improvement and 10 patients (18.9%) reported worsening of their comfort. Symptoms disappeared spontaneously in only 2 patients (3.7%), with no given treatment. In our study, 41 patients did not improve, 35 reported partial improvements, 12 worsened and 3 showed spontaneous remission of their symptomatology. Although our results are similar to those from Sardella et al., (23) our study reflects a higher number of patients with partial improvements.

Our analysis confirms that more than 58% of the patients are in never-ending struggles to find drugs to alleviate their symptoms. Moreover, these patients are great consumers of the sanitary resources. These numbers could exceed 75%, since the improvements reported are only partial.

In this study, we have analyzed a group of 91 patients suffering BMS symptoms. From the results of our analysis, we can con-clude that for a small number of patients, symptoms can remit spontaneously, within 5 years after the onset of BMS. Furthermore, only partial improvements were achieved in 38,4% of the patients having different treatments (e.g., topical clonazepam and mouth washes). This improvement is higher (up to 61,7%) when patients include topical clonazepam in their treatment. Nevertheless, in most cases, these improvements are closely related to psychotherapy.

It should be considered that the sample of patients in our study is not particularly large and that the time they were subject to controls was more than 18 months but not achieving, in some instances, the 5 years that the patients are subject to controls in other studies. Therefore the differences in the results may result from the smaller control time of the clinical evolution. We believe it is necessary to continue investigating this matter with larger populations and with controls during longer periods.

It is also necessary to carry on the investigation of the aetiopatogeny mechanisms of this disease to find a curative treatment.
